# Cinnamic acid derivatives as potential matrix metalloproteinase-9 inhibitors: molecular docking and dynamics simulations

**DOI:** 10.5808/gi.22077

**Published:** 2023-03-31

**Authors:** Mohammad Hossein Malekipour, Farzaneh Shirani, Shadi Moradi, Amir Taherkhani

**Affiliations:** 1 Dental Students Research Center, School of Dentistry, Isfahan University of Medical Sciences, Isfahan 8174673461, Iran; 2 Dental Research Center, Dental Research Institute, Department of Operative Dentistry, School of Dentistry, Isfahan University of Medical Sciences, Isfahan 8174673461, Iran; 3 Department of Medical Immunology, School of Medicine, Hamadan University of Medical Science, Hamadan 6517838678, Iran; 4 Research Center for Molecular Medicine, Hamadan University of Medical Sciences, Hamadan 6517838678, Iran

**Keywords:** Alzheimer′s disease, cancer, cinnamic acid, docking, inhibitor, MMP-9

## Abstract

Matrix metalloproteinase-9 (MMP-9) is a zinc and calcium-dependent proteolytic enzyme involved in extracellular matrix degradation. Overexpression of MMP-9 has been confirmed in several disorders, including cancers, Alzheimer′s disease, autoimmune diseases, cardiovascular diseases, and dental caries. Therefore, MMP-9 inhibition is recommended as a therapeutic strategy for combating various diseases. Cinnamic acid derivatives have shown therapeutic effects in different cancers, Alzheimer′s disease, cardiovascular diseases, and dental caries. A computational drug discovery approach was performed to evaluate the binding affinity of selected cinnamic acid derivatives to the MMP-9 active site. The stability of docked poses for top-ranked compounds was also examined. Twelve herbal cinnamic acid derivatives were tested for possible MMP-9 inhibition using the AutoDock 4.0 tool. The stability of the docked poses for the most potent MMP-9 inhibitors was assessed by molecular dynamics (MD) in 10 nanosecond simulations. Interactions between the best MMP-9 inhibitors in this study and residues incorporated in the MMP-9 active site were studied before and after MD simulations. Cynarin, chlorogenic acid, and rosmarinic acid revealed a considerable binding affinity to the MMP-9 catalytic domain (Δ*G*_binding_ < -10 kcal/mol). The inhibition constant value for cynarin and chlorogenic acid were calculated at the picomolar scale and assigned as the most potent MMP-9 inhibitor from the cinnamic acid derivatives. The root-mean-square deviations for cynarin and chlorogenic acid were below 2 Å in the 10 ns simulation. Cynarin, chlorogenic acid, and rosmarinic acid might be considered drug candidates for MMP-9 inhibition.

## Introduction

Salivary and genetic factors, interactions between the tooth structure, the microbial biofilm that has developed on the tooth surface, and carbohydrates are all factors in dental caries. Interactions between the tooth structure and the microbial biofilm grown on the tooth surface occur in tooth decay [1]. Dental caries is a very prevalent yet curable oral condition. Although the precise pathophysiology of dental decay is not fully understood, it is thought that a reactive inflammatory mechanism is involved once dentinal penetration of decay develops due to oral plaque bacteria adhering to the tooth surface and building up in a vulnerable population [2]. Although the World Health Organization acknowledgment that dental caries continues to be a severe health issue in the majority of developed countries, where it affects 60%–90% of children and the great majority of adults, dental caries is still an ignored subject. It is believed that the frequency of dental caries has lately grown among children 2–5 years old globally, making this age range a worldwide strategic priority issue [1]. Less than 1% of the enamel comprises an organic matrix, mainly minerals [3].

On the other hand, dentin is less mineralized and includes 19%–20% organic material, primarily type I collagen. The dentin enamel junction (DEJ), where these two tissues, enamel, and dentin, firmly connect, is where this is the case. The remaining macromolecular proteins, such as type IV and type VII collagen, may be buried in the basement membrane due to mineralization occurring during the formation of the DEJ. Several type IV gelatinases, including matrix metalloproteinase (MMP)-2 and MMP-9, MMP-20, and kallikrein-related peptidase 4, have been shown to degrade enamel matrix and basement membrane proteins both before and during the maturation phase [4]. It has long been known that genetic factors play a part in the genesis of dental caries. According to previous reports, some genetic factors in enamel creation may impact the disease [5,6]. The production of enamel and dentine in rats’ teeth depends heavily on the MMP’s genes, and MMP-9 and MMP-20 are allegedly implicated in the advancement of white spot lesions and early childhood caries [7,8].

Cancer is a severe category of disorders that affects many different body areas, causes global mortality, and is characterized by abnormal cell proliferation. After heart disease, cancer is the leading cause of mortality worldwide. 9.6 million people died from cancer in 2018 alone [9]. There are about 200 different types of cancer, including those of the lung, skin, breast, prostate, colorectal, and stomach. High body mass index, alcohol and cigarette use, inactivity, inadequate consumption of fruits and vegetables, and other risk factors are among the most prevalent causes of cancer, including colon cancer connected to colitis. Cancer mortality is caused by hepatitis, the human papillomavirus (HPV), and tobacco use (around 22%), as well as hepatitis and HPV (up to 25%). Cancer research has concentrated chiefly on the overexpressed molecules linked to the survival and growth of cancer cells [10].

MMPs are proteolytic metalloenzymes that rely on zinc [11]. One of the most complicated types of matrix metalloproteinases is MMP-9. It may break down extracellular matrix (ECM) components, which play a significant role in pathophysiological processes. Several disorders, namely cardiovascular diseases, neurodegenerative diseases, central nervous system disorders, and various cancers, are linked to MMP-9 dysregulation and overexpression [12]. MMPs are classified into six types based on how specific they are to certain substrates, including collagenase, gelatinase, matrilysins, stromelysins, membrane-type MMPs, and other types MMPs [13]. As a result, controlling and inhibiting MMP-9 is a crucial therapeutic strategy for treating different illnesses, especially cancer [14]. There are several different mechanisms to control MMPs, including transcription and maintaining pro-enzyme activity. Tissue inhibitor of metalloproteinase (TIMP) inhibits the activity of MMPs. There are four categories of TIMPs: TIMP-1, TIMP-2, TIMP-3, and TIMP-4. Stimulation and suppression of MMPs are unbalanced, which is critical for cancer pathophysiology [15]. Many variables, including cytokines (interleukin-1 and -6), growth factors (transforming growth factor, tumor necrosis factor-α, platelet-derived growth factor, basic fibroblast growth factor), as well as several hormones, boost the expression of MMP [16]. They play significant roles in many physiological mechanisms: growth, wound healing, tissue remodeling, organ morphogenesis, and angiogenesis. Angiogenesis, the process by which new blood vessels are created from preexisting ones, is crucial for the growth and spread of tumors [17]. Prominent MMPs, such as gelatinase A (MMP-2) and gelatinase B (MMP-9), are essential for the proteolytic cascade-driven breakdown of the ECM during colon cancer spread [18]. Malignant tumors’ ability to invade and spread is thought to be aided by MMP-9, which is present in the plasma membrane and its secreted forms. Type IV collagen, fibronectin, and laminin are ECM and basement membrane components that can be broken down by MMP-9, which has a wide pH optimal range. Additionally, urokinase plasminogen activator, other latent proteinases, and angiogenic factors or cytokine receptors may all be activated by MMP-9, which facilitates invasion and metastases by binding to the receptors on tumor cells and in the soluble form of these proteins [19].

Researchers have looked at using natural products as a source of potential therapeutic candidates. Approximately 60% of the new medications created to treat cancer came from natural sources [20]. Fruits, entire grains, vegetables, honey, and plants like *Cinnamomum cassia* (Chinese cinnamon) and *Panax ginseng* all contain the essential component of cinnamic acid, a naturally occurring aromatic carboxylic acid [21]. According to research, cinnamic acids are said to possess anti-inflammatory [22], anticancer [23], antibacterial [24], neuroprotective [25], and antioxidant [26] effects. By providing electrons that combine with radicals to produce stable compounds, cinnamic acid breaks down chain reactions involving radicals. Additionally, it is used as a fragrant element in detergents, flavorings, cosmetics, and toiletries. Enzymatic phenylalanine deamination may produce cinnamic acid [27]. Researchers have described various examples of cinnamic acid derivatives, such as ferulic acid, curcumin, caffeic acid, p-hydroxycinnamic acid, coumaric acid, and chlorogenic acids. The existence of hydroxyl groups on the phenyl ring that are either free or methoxylated helps to distinguish these cinnamic acid analogs from one another [28]. Different cinnamic acid derivatives’ biological activities have been linked to the type and location of their affix groups. Research on the creation of therapeutic molecules based on cinnamic acid has been stimulated by drug resistance and the lack of curative remedies to control cancer, microbial growth, neurological diseases, etc., with few undesirable side effects [29]. Cinnamic acid derivatives are helpful against a variety of malignancies, including breast, colon, and lung cancers. Cinnamic compounds have been shown to have anti-tumor proliferation properties. Apoptosis is one method used by cinnamic acid derivatives, such as cinnamaldehyde, to eradicate cancerous cells [30,31].

The present study hypothesized that cinnamic acid and its derivatives might act as MMP-9 inhibitors. Therefore, AutoDock 4.0 program was used to assess the binding affinity of selected cinnamic acid derivatives to the MMP-9 active site. To further examine the resistance of docked postures between the components that showed strong binding affinities, a molecular dynamics (MD) simulation was executed, and interactions among top-ranked compounds and residues inside the MMP-9 catalytic site were studied.

## Methods

### MMP-9 and small molecules structure preparation

The three-dimensional structure of MMP-9 was achieved at 1.59 Å x-ray resolution from the Research Collaboratory for Structural Bioinformatics (PDB ID: 5I12 [32]), available at https://www.rcsb.org [33]. The 5I12 file included one polypeptide chain (named chain A) with 157 residues, a selective sugar-conjugated arylsulfonamide carboxylate water-soluble inhibitor (PDB ID: H27), six calcium, and four zinc ions. The H27 was removed from the MMP-9 structure before docking and MD simulations. Thereafter, energy minimization of the protein was carried out using Discovery Studio Client version 16.1.0.15350. The binding affinity of 14 small molecules with an MMP-9 active site was evaluated using the AutoDock 4.0 tool. These ligands were as follows: Cinnamic acid, 11 Cinnamic acid derivatives, and two positive control inhibitors of MMP-9, including H27 (PubChem ID: 137349476) and MMP-9 inhibitor I (PubChem ID: 21310926). The structure of the ligands was prepared following our previous research [34,35].

### Molecular docking analysis

The in-silico simulations were executed using a Windows-based PC with the following characteristics: RAM, 32 GB; CPU, Intel Core i7; and operating system, 64-bit. AutoDock 4.0, available at http://autodock.scripps.edu, and Discovery Studio Client version 16.1.0.15350 were used to perform molecular docking and MD simulations, respectively. AutoDock 4.0 uses a Lamarckian Genetic Algorithm to calculate binding affinity between the studied ligand and protonated receptor [36,37]. The Kollman charges and polar hydrogen atoms were added to the protein’s structure [38], and subsequently, the PDBQT files of small molecules and MMP-9 were prepared for docking operations [39].

The main residues inside the enzyme’s active site were indicated by analyzing the two-dimensional view of interactions among H27 and residues inside the MMP-9 active site. Accordingly, the interacting amino acids with H27 were found to be Leu187, Leu188, Ala189, His226, His236, and Tyr248. Zinc and calcium ions were also included in the docking box. The grid box options were set to spacing, 0.375 Å; X-dimension, 64; Y-dimension, 50; Z-dimension, 64; X-center, 18.502; Y-center, –16.047; Z-center, 15.916.

A total of 50 docks were simulated for each ligand. The lowest binding energy in the largest root-mean-square deviations (RMSD) table was considered the Δ*G*_binding_ value between the studies ligand and MMP-9 catalytic domain. This study assigned the compounds demonstrating Δ*G*_binding_ value below −10 kcal/mol as top-ranked cinnamic acid derivatives. Interaction modes between these ligands and residues within the MMP-9 active site were studied using the BIOVIA Discovery Studio Visualizer version 19.1.0.18287.

### Cross-validation study

A cross-validation study was performed using the Molecular Operating Environment 2008.10 (MOE) [40] for top-ranked MMP-9 inhibitors according to the Δ*G*_binding_ values calculated by the AutoDock software (Δ*G*_binding_ < -10 kcal/mol). The number of runs was set to 50 for each ligand, and the lowest energy from the docked models was compared with the results achieved from the AutoDock 4.0.

### MD simulations

Only compounds that demonstrated inhibition constant (Ki) values at the picomolar scale (pM) using the AutoDock tool and revealed considerable binding affinity to the MMP-9 active site using the MOE software were considered for MD analysis. Discovery Studio Client version 16.1.0.15350 was utilized to execute MD simulations with a timescale of 10 ns [41]. The MMP-9 complex with its most potent inhibitors was examined over the simulation to determine the RMSD of the backbone atoms and the root mean square fluctuation (RMSF).

### Ethical approval

The present study was approved by the Ethics Committee of Isfahan University of Medical Sciences, Isfahan- Iran (ethics no. IR.MUI.RESEARCH.REC.1400.539).

## Results

### Binding affinity between MMP-9, cinnamic acid derivatives, and control inhibitors

**Fig. 1.**
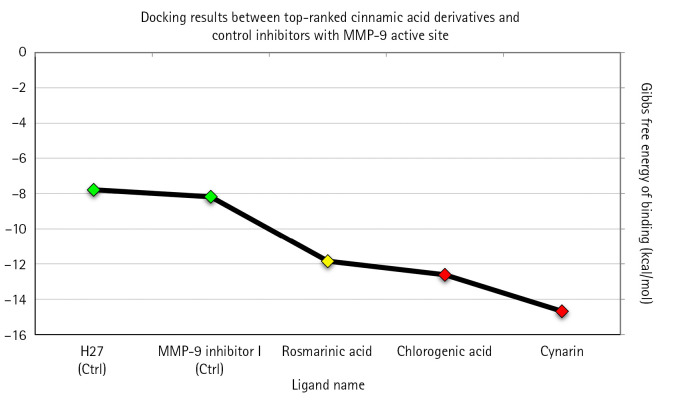
Binding affinity between this study’s top-ranked compounds, positive control inhibitors, and MMP-9 active site. The X-axis demonstrates the ligand name, and the Y-axis shows their corresponding ΔGbinding (kcal/mol). Green diamonds illustrate the control inhibitors, while the yellow and red spots show top-ranked inhibitors with Ki values at nanomolar and picomolar scales, respectively. MMP-9, matrix metalloproteinase-9; Ki, inhibition constant.

According to the molecular docking analysis, three compounds, including cynarin, chlorogenic acid, and rosmarinic acid, demonstrated Δ*G*_binding_ values < -10 kcal/mol and, therefore, were considered top-ranked cinnamic acids in this study. The Δ*G*_binding_ value between cynarin, chlorogenic acid, rosmarinic acid, and MMP-9 catalytic domain was calculated as -14.68, -12.62, and -11.85 kcal/mol, respectively. It was also estimated that cynarin and chlorogenic acid could bind to the MMP-9 active site at the picomolar concentration. Therefore, they were assigned as the most potent MMP-9 inhibitors from the studied cinnamic acid derivatives. The Ki value for cynarin and chlorogenic acid was calculated as 17.37 pM and 557.56 pM, respectively. The Δ*G*_binding_ value for MMP-9 inhibitor I and H27 were recorded as -8.18 and -7.79 kcal/mol, respectively. Table 1 demonstrates the Δ*G*_binding_ and Ki values for 12 cinnamic acid derivatives and two positive controls after docking with MMP-9 in this study. Moreover, intermolecular energy, internal energy, torisonal free energy, unbound system’s energy between top-ranked compounds and MMP-9 are presented in Table 2. Fig. 1 compares the Δ*G*_binding_ between top-ranked cinnamic acid derivatives, positive control inhibitors, and MMP-9 active site.

### Cross-validation

Cross-validation analysis was performed using the MOE tool to evaluate binding affinities between cynarin, chlorogenic acid, rosmarinic acid, and MMP-9 active site. Rosmarinic acid exhibited the most binding affinity to the MMP-9 active site, followed by cynarin and chlorogenic acid with docking scores of -14.8861, -13.6491, and -12.5566 kcal/mol, respectively (Table 3). Based on the docking results achieved from AutoDock 4.0 and MOE, cynarin and chlorogenic acid were assigned for further MD simulations. Of note, MD analyses were simulated based on the protein-ligand complexes achieved from AutoDock 4.0.

### Interaction mode analysis

**Fig. 2.**
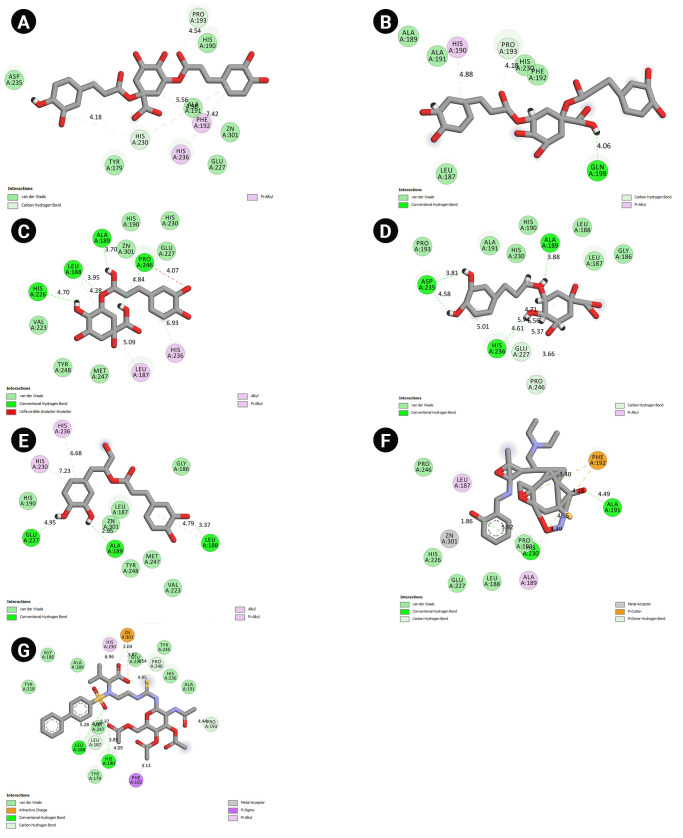
Interaction modes between amino acids inside the MMP-9 catalytic site, top-ranked cinnamic acid derivatives in this study, and positive control inhibitors. (A) Cynarin before MD simulation. (B) Cynarin after MD simulation. (C) Chlorogenic acid before MD simulation. (D) Chlorogenic acid after MD simulation. (E) Rosmarinic acid. (F) MMP-9 inhibitor I. (G) H27. MD, molecular dynamics; MMP-9, matrix metalloproteinase-9.

Interaction modes between cynarin, chlorogenic acid, rosmarinic acid, MMP-9 inhibitor I, H27, and residues within the MMP-9 active were visualized using the BIOVIA Discovery Studio Visualizer software. Furthermore, the interactions among cynarin, chlorogenic acid, and MMP-9 active site residues were analyzed after 10 ns MD simulations (Table 4). Chlorogenic acid showed the most considerable H-bonds with the residues within the MMP-9 active site before and after MD simulations. Before MD analysis, chlorogenic acid formed four classical hydrogen bonds with Leu188, Ala189, His226, and Pro246 inside the MMP-9 active site. After 10 ns MD simulation, this herbal component demonstrated four conventional H-bonds with Ala189, Asp235, His236, and two non-classical hydrogen bonds with Glu227 and Pro246 within the MMP-9 catalytic domain. A two-dimensional view of the interactions is presented in Fig. 2.

### MD simulations

**Fig. 3.**
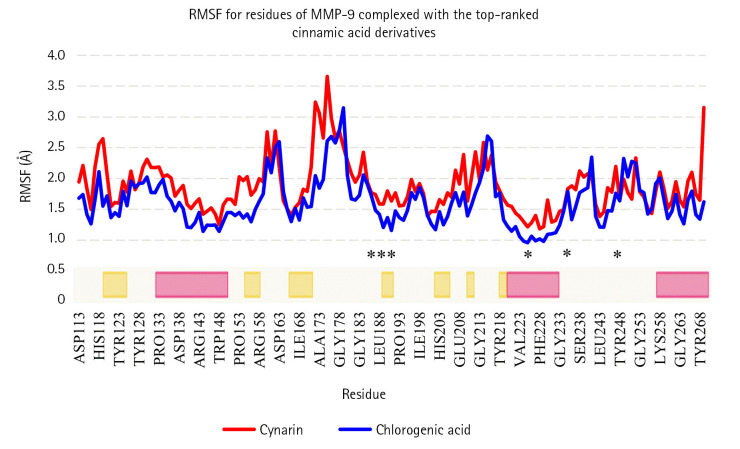
RMSF for backbone atoms of MMP-9 complexed with cynarin and chlorogenic acid. The secondary structure of MMP-9 was obtained from the RCSB PDB. Yellow and pink colors present β-strand and α-helix structures, respectively. X-axis illustrates the name of residue, while the Y-axis presents the RMSF. Residues incorporated inside the MMP-9 active site are marked with asterisks. RMSF, root mean square fluctuation; MMP-9, matrix metalloproteinase-9.

**Fig. 4.**
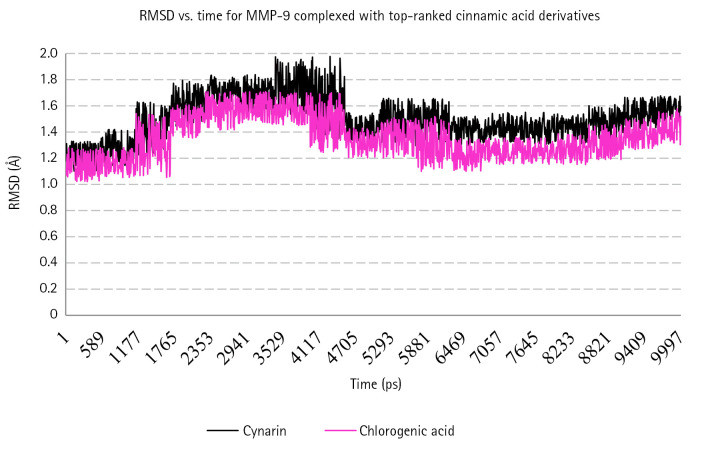
RMSD for backbone atoms of MMP-9 complexed with Cynarin and Chlorogenic acid. X-axis shows the simulation time, while the Y-axis demonstrates the RMSD. RMSD, root-mean-square deviations; MMP-9, matrix metalloproteinase-9.

**Fig. 5.**
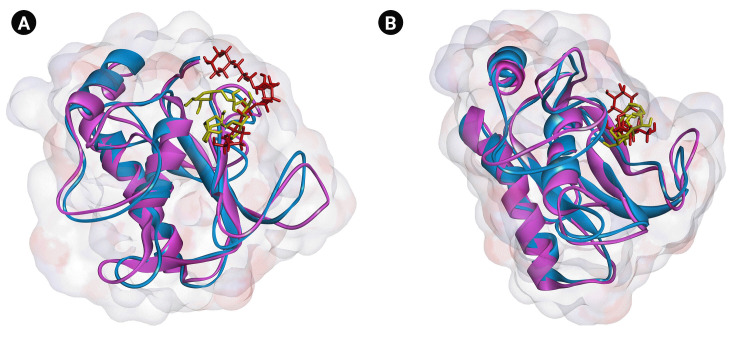
Superimposed structures of MMP-9 complexed with cynarin (A) and chlorogenic acid (B) before and after 10 ns MD simulations. Blue and violet colors show the protein before and after MD simulations. Yellow and red illustrate the small molecule before and after MD simulations. MMP-9, matrix metalloproteinase-9; MD, molecular dynamics.

Based on the MD analyses, the docked poses of cynarin and chlorogenic acid were stable in 10 ns simulations. For cynarin, it was observed that the range of RMSD was approximately between 1 and 2 Å. Besides, the range of RMSD for chlorogenic acid was calculated between 1 and 1.7 Å. However, the docked poses of both compounds were stable after 6 ns simulation. According to the molecular mechanics/generalized Born surface area analysis, the β-strand and α-helix structures demonstrated less fluctuation than the other secondary structures. Figs. 3 and 4 illustrate RMSF and RMSD for MMP-9 backbone atoms complexed with cynarin and chlorogenic acid, respectively. Fig. 5 illustrates the superimposed structures of MMP-9 complexed with cynarin and chlorogenic acid before and after 10 ns MD simulations.

## Discussion

Virtual screening and computer-aided medication design are essential for discovering new core compounds. This combinatorial strategy allows us to reduce trial time and cost by choosing a more targeted biological target [42,43]. Based on our findings, cynarin, chlorogenic acid, and rosmarinic acid demonstrated a salient binding affinity to the MMP-9 active site. These components were also found to be more closely bound to the enzyme than MMP-9 Inhibitor I and H27, which are the enzyme’s recognized inhibitors.

Cynarine is a caffeoylquinic acid molecule isolated from the Traditional Chinese Medicine artichoke (*Cynara scolymus* L.) that possesses anti-obesity and antioxidant liver properties in high-fat diet-induced obese mice [44]. With a binding energy of -14.68 kcal/mol in this investigation, cynarin had the highest binding affinity of all the mentioned cinnamic acid derivatives. Elmosallamy’s research aimed to determine the synergistic impact and function of artichoke extract (receptacle and bracts) in the therapy of hepatocellular carcinoma (HCC). They found that artichokes contain polyphenols such as cynarin, caffeoylquinic acid, chlorogenic acid, flavonoids including luteolin, and glycosides. Thioacetamide produced liver damage, which was demonstrated by a considerable rise in the levels of the liver enzyme markers alanine aminotransferase, aspartate aminotransferase, and alkaline phosphatase, as well as total protein activity in the blood. The total bilirubin function levels revealed a notable drop in albumin capacity and a considerable decrease in bilirubin levels in the plasma. Additionally, it increased the activities of the metalloproteinases MMP-3, MMP-9, and MMP-12 compared to the control values that demonstrated oxidative stress. In contrast to the HCC group, artichoke treatment dramatically decreased the rise of liver enzymes and oxidative stress. It also caused apoptosis via inhibiting metalloproteinase [45]. A decrease in the growth of MCF-7 breast cancer cells was shown in *in vitro* research using Echinacea purpurea hexane components. At the same time, cynarin from the roots of Echinacea Angustifolia also demonstrated an antiproliferative effect. The researchers also asserted that cynarin improved doxorubicin’s cytotoxic effects on MCF-7 breast cancer cells [46]. The artichoke, or *Cynara scolymus* L. (Asteraceae), has many medicinal actions, including anticancer, antitoxin, hypolipidemic, choleretic, antioxidant, anti-inflammatory, and hepatoprotective characteristics [47].

One of the most readily accessible phenolic acid components in foods, including coffee and tea, is chlorogenic acid [48]. A study team confirmed that the bioactive coffee chemical components Trigonelline and chlorogenic acids, primarily contained in *C. canephora*, had antibacterial action toward these cariogenic bacteria [49,50]. The ability of *S. mutans* to adhere to saliva-coated hydroxyapatite beads is inhibited by the substances trigonelline, caffeine, and chlorogenic acid found in green and roasted coffee. Studies on the green, oolong, and black tea suggest that the polyphenols in these beverages have an anti-caries impact via an anti-microbial mechanism of action and that the galloyl esters of (−)-epicatechin, (−)-epigallocatechin, and (−)-gallocatechin have increasing antibacterial activity. The anti-cariogenic properties of cocoa, coffee, and tea polyphenols against α-hemolytic streptococci point to the need for more research into the potential role of these drinks in preventing the etiology of dental caries [51]. Numerous studies have shown that chlorogenic acid affects type 2 diabetes and obesity’s metabolic characteristics via mechanisms like the AMP-activated protein kinase (AMPK) pathway. Additionally, this chemical can slow the development of cancer cells by primarily disrupting their metabolic processes [52].

Abnormalities cause most cancers in the epidermal growth factor receptor (EGFR)/phosphatidylinositol 3-kinase (PI3K)/mammalian target of rapamycin pathway, hypoxia-inducible factor (HIF), and vascular endothelial growth factor (VEGF) expression. The main trigger for HIF-1 overexpression is hypoxia. Additionally, the PI3K pathway and EGFR have a role in the overexpression of HIF-1. As a result, cancer treatment’s primary focus is on this route’s elements. Chlorogenic acid may downregulate the HIF-1/AKT pathway to prevent hypoxia-induced angiogenesis in HUVEC cells. Chlorogenic acid inhibited VEGF-induced angiogenesis in vivo by preventing AKT activation [53]. In addition, VEGF expression and release were downregulated in chlorogenic acid–treated cells compared to hypoxia only. According to Park et al. [54], HIF-1 transcriptional activation was lowered by chlorogenic acid treatment (2 or 10 mM). We discovered that chlorogenic acid, a derivative of cinnamic acid, had the second-highest affinity, with a binding energy of -12.62 kcal/mol. Although the molecular targets of the chlorogenic acid molecule are unknown, cancer cells stimulated with the molecule displayed differential expression of transcriptional factors and regulatory molecules. The maximum standard substructure score obtained by superimposing the biophoric components of chlorogenic acid and curcumin is 0.90.

Caffeic acid and 3,4-dihydroxy phenyl lactic acid are esterified to form rosmarinic acid. It is often discovered in species of the Lamiaceae subgroup Nepetoideae and Boraginaceae. However, it may also be found in specific fern, hornwort species, and other species of higher plant groups. The intriguing biological effects of rosmarinic acid include its antiviral, antibacterial, anti-inflammatory, and antioxidant properties [55]. In the current study, the binding energy of rosmarinic acid was calculated to be -11.85 kcal/mol, making it the third most reactive of all cinnamic acid derivatives in this study. Radziejewska et al. [56] reported that rosmarinic acid inhibited MMP-9 activity, which is linked with increased production of collagen type I, the primary ECM substrate destroyed by MMPs. In Han’s research, cell cycle delay and death were two mechanisms of rosmarinic acid stifling colorectal cancer (CRC) cell growth. When rosmarinic acid was administered to CRC cells, the expressions of MMP-2 and MMP-9 were reduced, and the cells’ ability to invade and migrate was suppressed. The expression of adhesion molecules such as intercellular adhesion molecule-1 and integrin β1 was similarly decreased by rosmarinic acid therapy. For instance, AMPK activation accounted for the impacts of rosmarinic acid on epithelial–mesenchymal transition and MMPs expressions. Furthermore, in a mouse model, rosmarinic acid blocked AMPK activation and reduced lung metastasis of CRC cells [57]. In another study, An et al. [58] MMP-2, MMP-9, PI3K, AKT, nuclear factor κB (NF-κB), and apoptosis-related proteins Bax, Bcl-2, and cleaved caspase-3 protein expression were all identified. MMP-2 and MMP-9 expression levels were reduced, migration and infiltration were prevented, and proliferation rates were all noticeably diminished by rosmarinic acid. Terminal deoxynucleotidyl transferase dUTP nick end labeling labeling demonstrated that rosmarinic acid increased HepG2 cell death dose-dependently. According to this research, the expression of the pro-apoptotic proteins Bax and cleaved caspase-3 was upregulated, whereas that of the apoptosis inhibitor protein Bcl-2 was downregulated. Additionally, they discovered that rosmarinic acid-mediated suppression of HepG2 cell metastasis included the PI3K/Akt/NF-κB signaling pathway [58].

In conclusion, the present study suggests that cynarin, chlorogenic acid, and rosmarinic acid have a salient binding affinity to the MMP-9 active site (Δ*G*_binding_ < -10 kcal/mol). Also, it was found that cynarin and chlorogenic acid potentially can block the MMP-9 catalytic site at the picomolar scale. The docked poses for cynarin and chlorogenic acid were stable in 10 ps simulation. Cynarin, chlorogenic acid, and rosmarinic acid may be well suited for developing preventative medicines against several human disorders such as cancers, Alzheimer′s disease, autoimmune diseases, cardiovascular diseases, and tooth decay. In future research, it is suggested to perform MD simulations for a much more extended period. After that, the conformational changes of MMP-9 and interactions of ligands with MMP-9 could be examined using fluorescence spectroscopy [59]. Tryptophan (Trp), phenylalanine (Phe), and tyrosine (Tyr) are responsible for the emission of proteins [60]. In this regard, small molecules act as quenchers when they are located near Trp and/or Tyr [61]. According to the present results, Tyr179 and Tyr248 are located within the MMP-9 active site. Therefore, fluorescence spectroscopy might be suitable for MMP-9 ligand binding analysis in-vitro. Subsequently, the therapeutic effects of cynarin, chlorogenic acid, and rosmarinic acid could be evaluated in animal models and human clinical trials.

## Figures and Tables

**Table 1.** Binding energy and *K*i values between cinnamic acid derivatives and two MMP-9 inhibitors after docking with MMP-9

Ligand ID	Ligand name	Binding energy (kcal/mol)	*K*i
6124212	Cynarin	–14.68	17.37 pM
1794427	Chlorogenic acid	–12.62	557.56 pM
5281792	Rosmarinic acid	–11.85	2.06 nM
5281787	Caffeic acid phenethyl ester	–8.61	486.30 nM
5281759	Caffeic acid 3-glucoside	–8.14	1.09 μM
5372945	N-p-Coumaroyltyramine	–8.06	1.24 μM
689043	Caffeic acid	–6.92	8.42 μM
444539	Cinnamic acid	–6.90	8.76 μM
637775	Sinapinic acid	–6.68	12.66 μM
637540	o-Coumaric acid	–6.44	18.96 μM
445858	Ferulic acid	–6.33	22.88 μM
637542	p-Coumaric acid	–5.72	64.31 μM
21310926	MMP-9 Inhibitor I (Ctrl)	–8.18	1.02 μM
137349476	H27 (Ctrl)	–7.79	1.94 μM

*K*i, inhibition constant; MMP-9, matrix metalloproteinase-9.

**Table 2.** Details of binding energies between matrix MMP-9, top-ranked cinnamic acids, and MMP-9–positive control inhibitors

Ligand name	Intermolecular energy (kcal/mol)	Total internal energy (kcal/mol)	Torisonal free energy (kcal/mol)	Unbound system's energy (kcal/mol)	Free binding energy (kcal/mol)
Cynarin	–6.4	–16.62	6.26	–2.08	–14.68
Chlorogenic acid	–7.34	–10.99	4.18	–1.53	–12.62
Rosmarinic acid	–8.11	–9.24	4.47	–1.02	–11.85
MMP-9 inhibitor I (Ctrl)	–9.89	–2.09	2.09	–1.72	–8.18
H27 (Ctrl)	11.73	–4.96	5.67	–3.23	–7.79

MMP-9, matrix metalloproteinase-9.

**Table 3.** MOE docking score (kcal/mol) of top-ranked cinnamic acids based on the AutoDock 4.0, against MMP-9 active site (PDB ID: 5I12)

Ligand name	E_conf	E_place	E_score1	E_refine	E_score2
Cynarin	1.2117	–47.5798	–8.8624	–13.1294	–13.6491
Chlorogenic acid	0.4	–57.0131	–8.2766	0.6026	–12.5566
Rosmarinic acid	1.268	–59.0988	–8.271	34.3737	–14.8861

MMP-9, matrix metalloproteinase-9.

**Table 4.** Interactions between MMP-9 catalytic site, top-ranked cinnamic acid derivatives, and MMP-9 control inhibitors

Ligand name	Hydrogen bond (distance A)	Hydrophobic interaction (distance A)	Miscellaneous (distance A)	Electrostatic (distance A)
Cynarin (before MD)	His230 (4.18: Non-Classical); Pro193 (4.54: Non-Classical)	His236 (7.42: Pi/Alkyl); His230 (4.98: Pi/Alkyl); Phe192 (5.56: Pi/Alkyl)	NA	NA
Cynarin (after MD)	Gln199 (4.06: Classical); Pro193 (4.18: Non-Classical)	His190 (4.88: Pi/Alkyl)	NA	NA
Chlorogenic acid (before MD)	His226 (4.70: Classical); Leu188 (3.95: Classical); Ala189 (3.70: Classical); Pro246 (4.84: Classical)	Leu187 (5.09: Alkyl); Leu188 (4.28: Alkyl); His236 (6.93: Pi/Alkyl)	NA	NA
Chlorogenic acid (after MD)	Asp235 (3.81: Classical, 4.58: Classical); His236 (4.61: Classical); Ala189 (3.88: Classical); Glu227 (4.71: Non-Classical); Pro246 (3.66: Non-Classical)	His236 (6.54: Pi/Alkyl)	NA	NA
Rosmarinic acid	Glu227 (4.95: Classical); Ala189 (2.95: Classical); Leu188 (3.37: Classical)	Leu188 (4.79: Alkyl); His230 (7.23: Pi/Alkyl); His236 (6.68: Pi/Alkyl)	NA	NA
MMP-9 Inhibitor I (Ctrl)	Ala191 (4.49: Classical); His230 (4.49: Non-Classical, 4.55: Non-Classical)	Ala189 (5.67: Pi/Alkyl); Leu187 (5.92: Pi/Alkyl); Phe192 (4.55: Pi/Alkyl); Tyr179 (3.99: Pi/Alkyl, 4.08: Pi/Alkyl)	Phe192 (3.40: Pi-Cation, 4.18: Pi-Cation)	His230 (4.49: Sulfur); Zn (1.86: Metal)
H27 (Ctrl)	His190 (3.85: Classical); Leu188 (4.99: Classical); Leu187 (3.37: Non-Classical); Pro193 (4.44: Non-Classical); Pro246 (4.54: Non-Clasical, 4.95: Non-Classical)	NA	Zn (3.82: Attractive Charge)	Zn (2.04: Metal)

MMP-9, matrix metalloproteinase-9; MD, molecular dynamics; NA, not available.
